# Current Concepts in Frontal Sinus Fracture Management

**DOI:** 10.3390/cmtr19020021

**Published:** 2026-04-08

**Authors:** Tsung-yen Hsieh, Mary Roz Timbang, Edward Bradley Strong

**Affiliations:** 1 Department of Otolaryngology—Head & Neck Surgery, University of Southern California, Los Angeles, CA 91411, USA; 2 Department of Otolaryngology—Head & Neck Surgery, Children’s Hospital Los Angeles, Los Angeles, CA 91411, USA; mtimbang@gmail.com; 3 Department of Otolaryngology, University of California, Davis, Sacramento, CA 95817, USA

**Keywords:** frontal sinus, frontal sinus fracture, craniomaxillofacial fracture, sinus surgery, cranialization

## Abstract

Frontal sinus fractures typically reflect high-energy trauma and must be evaluated and treated carefully to avoid long-term problems including contour deformity, sinus dysfunction, cerebrospinal fluid (CSF) leakage, chronic sinusitis, and mucocele formation. This article outlines frontal sinus anatomy, diagnostic pathways, and evolving treatment concepts in detail. An anatomically driven treatment algorithm is emphasized, with a focus on preservation of sinus function whenever possible and preference for conservative management. Advanced procedures, such as endoscopic sinus surgery and cranialization, are reviewed in the context of managing more severe injuries. Key points: (1) Clinical decision-making in the management of frontal sinus fractures is best guided by evaluating the status of the anterior table, posterior table, and nasofrontal outflow tract, with treatment options ranging from nonoperative care to open or endoscopic surgery. (2) Improvements in endoscopic techniques, combined with evidence supporting less aggressive strategies, have shifted management toward more conservative approaches, reserving open procedures for higher-grade injuries. (3) Extended follow-up is essential to identify delayed problems such as mucoceles, chronic sinusitis, frontal bone osteomyelitis, and contour irregularities.

## 1. Introduction

Frontal sinus fractures usually arise after high-velocity impacts, including motor vehicle collisions, interpersonal violence, and sports-related trauma. These injuries comprise approximately 5–15% of all facial fractures [1]. If not addressed appropriately, they may lead to facial disfigurement, chronic sinusitis, mucocele, intracranial infection, or even death, with many of these complications presenting months or years after the initial trauma [2,3].

Although facial fracture management has advanced substantially, there is still no universal agreement on the ideal approach to frontal sinus trauma. In preparing this narrative review, we drew primarily on our institutional experience in the management of frontal sinus fractures and supplemented this with a targeted review of the contemporary literature, using database searches and citation tracking of key references. This article discusses current strategies for diagnosis and treatment and proposes an anatomically based algorithm to guide management.

## 2. Anatomy

The frontal sinus is not present at birth; pneumatization generally begins around 2 years of age, when anterior ethmoidal cells extend into the frontal bone. The sinus typically becomes radiographically visible by about age 8 and achieves adult dimensions in early adolescence, although the ultimate size and shape vary widely among individuals. Roughly 4% of adults lack a frontal sinus, about 5% have only rudimentary air cells, and approximately 10% demonstrate a unilateral sinus [2].

**Figure 1:**
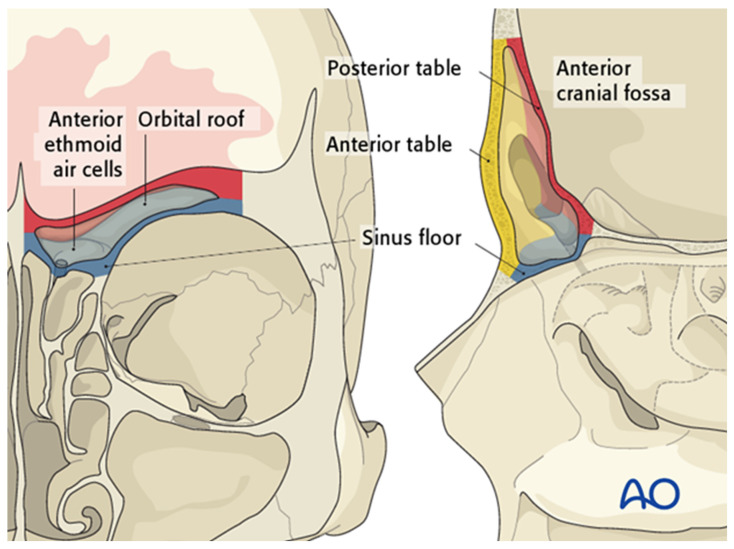
The frontal sinus demonstrates several key anatomic relationships: the sinus floor (blue) corresponds to the orbital roof and anterior ethmoid air cells; the posterior table (red) abuts the anterior cranial fossa; and the anterior table (yellow) defines the frontal contour. Reproduced with permission from AO Foundation.

**Figure 2:**
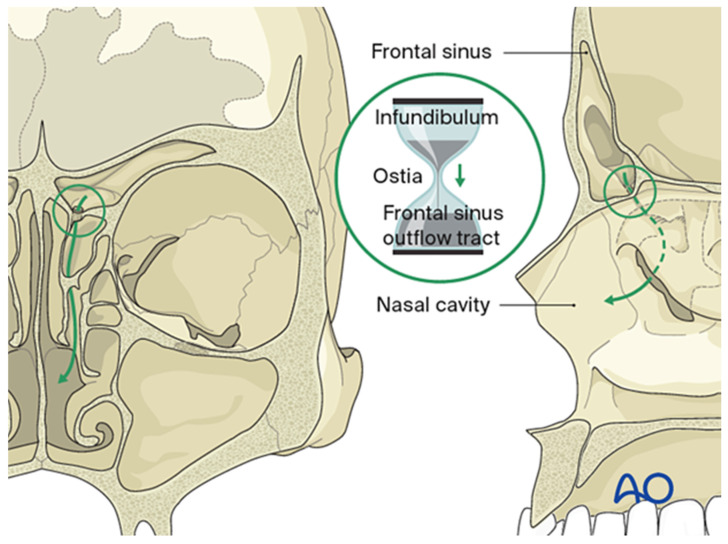
The frontal sinus drains through an hourglass-shaped outflow tract into the ethmoid sinus and nasal cavity, with a true ostium 3–4 mm in diameter at its narrowest point. Reproduced with permission from AO Foundation.

Key anatomic relationships include the orbital roof (forming the sinus floor), the anterior cranial fossa (forming the posterior sinus wall), and the overlying forehead and glabella (constituting the anterior wall) (Figure 1). The anterior table is relatively thick (4–12 mm) and more substantial than the posterior table, which measures approximately 0.1–4.8 mm. The frontal sinus drainage pathway is characteristically hourglass-shaped; the true ostium represents the narrowest segment, measuring 3–8 mm in diameter, with the sinus infundibulum superior to the ostium and the frontal recess inferiorly (Figure 2) [2,3]. The ostium is bounded anteriorly by the agger nasi cell, posteriorly by the anterior ethmoid artery, and laterally by the orbit [4].

## 3. Clinical Presentation and Assessment

Initial assessment should follow standard trauma principles, with attention to airway, breathing, circulation, disability, and exposure (ABCDE). A detailed head and neck examination is required, noting lacerations over the glabella or supraorbital ridge, significant edema, or any visible depression of the frontal bone. Concomitant facial fractures are common, especially orbital and naso-orbito-ethmoid injuries, and should be carefully evaluated [1].

Symptoms such as paresthesia and epistaxis should be documented, and any clear rhinorrhea must prompt evaluation for possible CSF leakage. When suspicion for a leak is high, fluid should be tested for Beta-2 transferrin, which has a reported specificity of 99% and sensitivity of 97% [5].

## 4. Radiological Evaluation

Thin-cut (0.625 mm) computed tomography (CT) remains the gold standard for diagnosing frontal sinus fractures. Axial, coronal, sagittal, and three-dimensional (3D) reconstructions should be obtained to fully appreciate the pattern of injury. Axial images are especially helpful in evaluating the anterior and posterior tables, whereas coronal and sagittal views assist in assessing the frontal outflow tract and skull base [6].

Three-dimensional reconstructions improve fracture visualization and surgical planning by clarifying fragment size, shape, orientation, and potential surgical corridors, and they may limit the extent of dissection required. These reconstructions are also useful for patient counseling, with Wickwire et al. demonstrating improved patient understanding and information retention when 3D images are used [6].

## 5. Diagnosis and Treatment Planning

Historically, early open repair of frontal sinus fractures was favored to reduce the risk of intracranial complications. With the evolution of endoscopic techniques, however, treatment has shifted toward a more conservative, “expectant” approach. In this framework, management strategies are divided into *primary repair* versus *expectant management* with secondary intervention as needed, typically for functional problems (such as mucocele or chronic sinusitis) or aesthetic deformity.

**Figure 3:**
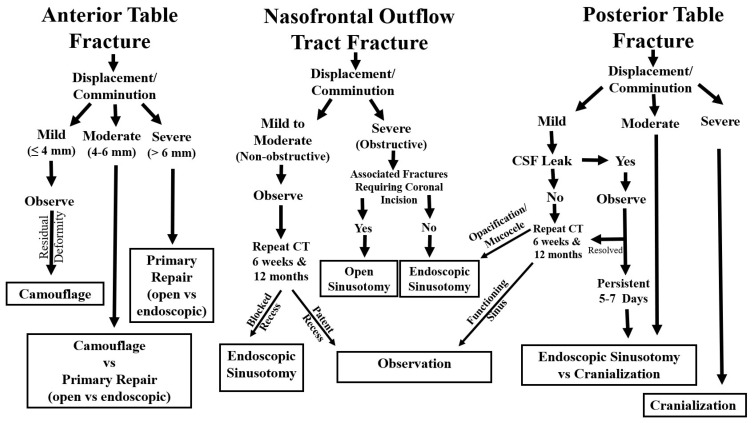
Algorithm illustrating the treatment of frontal sinus fractures based on anterior table, posterior table, and nasofrontal outflow tract involvement.

Treatment selection hinges on assessing three key elements: (1) the anterior table, (2) the posterior table and dural integrity (including CSF leakage), and (3) the nasofrontal outflow tract (Figure 3). While restoration of normal sinus physiology may not always be achievable, the overarching goals are to establish a safe sinus environment and restore an acceptable frontal contour.

### 5.1. Anterior Table Fractures

Anterior table fractures seldom lead to significant sinus dysfunction, so aesthetic considerations typically dominate decision-making. Early soft tissue swelling can obscure underlying contour deformities, and minor asymmetries may remain unnoticed or unimportant to patients even after edema resolves, complicating the decision to intervene surgically [7,8,9]. The authors classify these injuries as mild (less than 4 mm displacement), moderate (4–6 mm), or severe (greater than 6 mm), with different management strategies for each category (Figure 3).

- ***Mild displacement (<4 mm)***

Multiple studies support a conservative approach in the setting of mild displacement. Kim et al. reviewed 51 patients with anterior table fractures, 44 of whom had mild displacement, and none developed a contour deformity [9]. Dalla Torre et al. reported that among 91 patients with less than 5 mm displacement, only four developed contour irregularities, and none requested surgical correction [10]. Accordingly, observation is typically favored, with camouflage procedures reserved for patients who later develop clinically meaningful deformities.

- ***Moderate displacement (4–6 mm)***

Evidence regarding optimal management of moderately displaced fractures is limited. Primary repair may be considered if it can be performed through an existing laceration or a well-camouflaged upper blepharoplasty incision without substantial added morbidity. In other cases, expectant management is preferred, allowing patients to decide on surgery after edema resolves; in the authors’ experience, approximately 75% of patients ultimately forego operative correction.

- ***Severe displacement (>6 mm, large area, severe comminution)***

Severely displaced fractures, particularly those involving large areas or marked comminution, carry a higher risk of noticeable contour deformity. Although observation is possible, primary repair is usually recommended in this setting [8]. Some surgeons offer endoscopic repair, but this approach should be considered in light of surgeon expertise and potential complications [11].

### 5.2. Posterior Table Fractures and Dural Injury

Traditional recommendations for posterior table fracture repair have included displacement greater than 2 mm or more than the width of the table [12]. However, these thresholds are not strongly supported by contemporary evidence, and more recent series favor conservative management with close follow-up [13,14]. The authors categorize posterior table injuries as mild, moderate, or severe (Figure 3).

- ***Mild injuries (0–4 mm displacement, mild pneumocephalus, mild comminution)***

In the absence of a CSF leak, mild posterior table injuries are treated medically with topical nasal steroids and saline irrigations initiated 1–3 weeks post-injury. Follow-up CT scans are obtained at approximately 6 weeks and 12 months, or sooner if clinically warranted; continued medical therapy is advised for persistent mucosal thickening. Progression of symptoms, sinus opacification, or mucocele formation are indications for endoscopic frontal sinusotomy.

When a CSF leak is present, skull base precautions are instituted and the patient is observed for 5–7 days, as most leaks will resolve spontaneously [15]. Persistent leakage requires surgical repair, typically via an endoscopic approach; if this is not feasible, open cranialization of the frontal sinus is indicated.

- ***Moderate injuries (4–6 mm displacement, moderate pneumocephalus, moderate comminution)***

Moderately displaced posterior table fractures usually warrant operative repair regardless of CSF leak status. The authors favor endoscopic repair whenever safely achievable [16]. If endoscopic access is limited by technical or anatomic constraints, open cranialization of the sinus is recommended.

- ***Severe injuries (>6 mm displacement, large area, severe comminution and pneumocephalus)***

Severe posterior table injuries, particularly those with extensive displacement, comminution, and pneumocephalus, almost always require open repair with frontal sinus cranialization.

### 5.3. Frontal Sinus Outflow Tract Fractures

Historically, damage to the frontal sinus outflow tract was thought to inevitably lead to mucocele formation and was treated with sinus obliteration. Current data suggest that 80–90% of sinuses with mild to moderate tract injury will re-aerate when managed conservatively [17,18]. If secondary problems such as sinusitis or mucoceles develop, they can be addressed with endoscopic frontal sinusotomy (Draf III), which has been shown to safely and effectively treat even established mucoceles [19]. The authors classify these injuries as non-obstructive versus obstructive, based on the degree of outflow tract compromise (Figure 3).

- ***Non-obstructive injuries***

**Figure 4:**
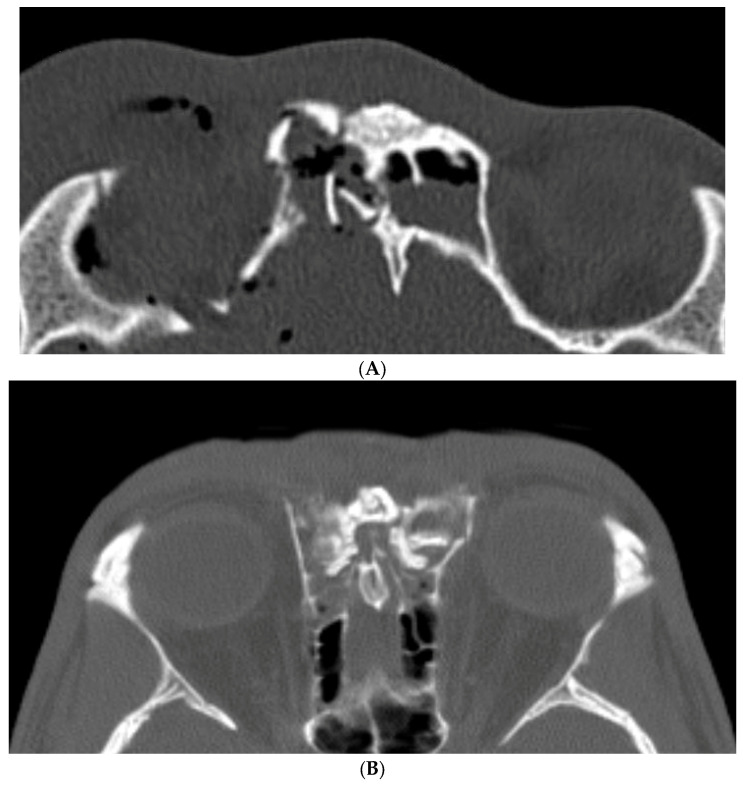
(**A**) Non-obstructive frontal sinus outflow tract fracture. (**B**) Obstructive frontal sinus outflow tract injury.

Non-obstructive injuries are defined by partial damage without complete occlusion of the frontal outflow tract (Figure 4A). These cases have high rates of spontaneous sinus re-aeration (80–90%) with expectant care, which typically includes topical nasal steroids and saline irrigations initiated 1–3 weeks after trauma. Routine surveillance CT scans are obtained at 6 weeks and 12 months or as clinically indicated; when the sinus re-aerates without significant mucosal thickening, no further treatment is necessary. Persistent or worsening mucosal thickening, progressive symptoms, or frank opacification are indications for endoscopic frontal sinusotomy (Draf III) [12].

- ***Obstructive injuries***

Obstructive injuries involve complete collapse or occlusion of the outflow tract (Figure 4B). These typically merit primary surgical intervention. The authors favor maintaining sinus function via endoscopic frontal sinusotomy rather than obliteration, which also enables postoperative endoscopic surveillance and reduces the risk of iatrogenic complications and long-term mucocele formation [16]. When patients also require a coronal approach for associated injuries (such as naso-orbito-ethmoid fractures), a frontal sinusotomy can be performed from above as an “open Draf III”.

When nasofrontal recess injury occurs in combination with posterior table fractures and cerebrospinal fluid (CSF) leak, management should consider both the integrity of the outflow tract and the risk of intracranial complications. Minimally displaced posterior table fractures with a transient CSF leak and a patent outflow tract may be observed with close clinical and radiologic follow-up, whereas significantly displaced or comminuted fractures with a persistent leak and clear FSOT obstruction may require operative repair of the dural defect combined with frontal sinus cranialization. Our proposed algorithm therefore escalates from observation and endoscopic FSOT-preserving techniques to cranialization as the severity of posterior table displacement, FSOT injury, and CSF leak persistence increases.

## 6. Surgical Techniques

**Fracture camouflage**

If anterior table fractures are observed or present secondarily, any residual anterior table contour defects can be addressed using alloplastic implants. Access can be obtained via existing lacerations, forehead rhytids, or an upper blepharoplasty incision for low frontal defects. The authors prefer an endoscopic brow lift approach which affords broad exposure to most of the anterior table. Once the defect is exposed, the implant—often porous polyethylene sheeting, or a patient-specific implant (e.g., titanium, porous polyethylene, or polyether-etherketone)—is used to camouflage the depression [20].

**Open reduction and internal fixation**

**Figure 5:**
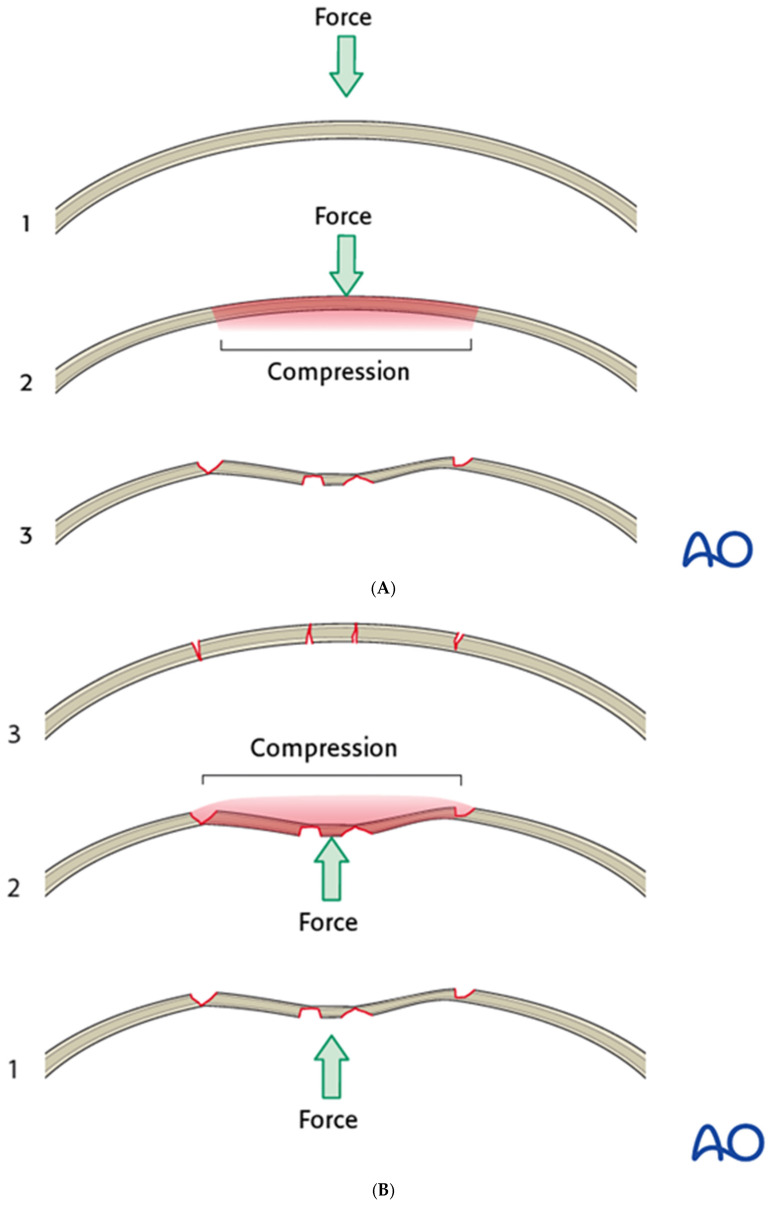
(**A**) The normal anterior table is convex; compressive forces convert this into a concavity. Reproduced with permission from AO Foundation. (**B**) Significant force may be needed to mobilize depressed fragments and overcome residual compressive forces. Reproduced with permission from AO Foundation.

Open reduction and internal fixation (ORIF) of anterior table fractures is commonly performed through a coronal incision or existing lacerations, providing excellent exposure but sometimes challenging reduction. Because the convex frontal bone first experiences compression and then becomes concave, substantial force may be required to restore the original convex contour (Figure 5A,B). Techniques include placing a bone hook into the fracture line to mobilize fragments, inserting a mini-screw into a depressed segment and using a clamp to apply controlled traction, or removing a fragment to relieve tension, all while avoiding excessive force that could avulse the segment.

**Figure 6:**
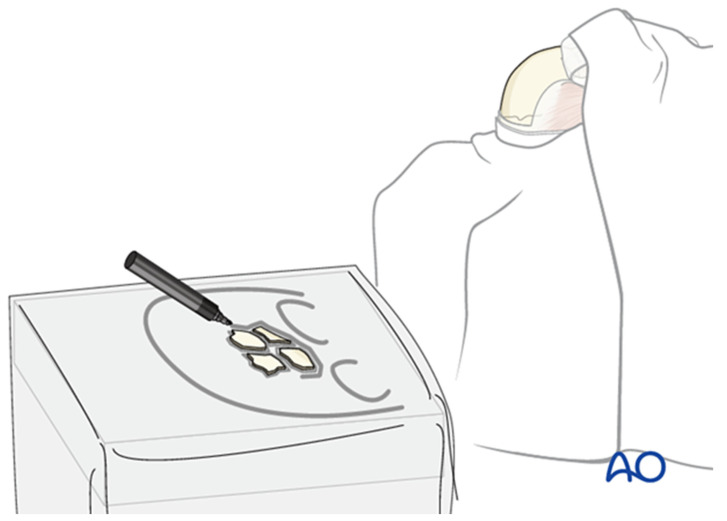
Removed anterior table fragments are placed on a simple sketch of the skull to preserve their orientation and outline the overall defect; bone is kept moist until reimplantation. Reproduced with permission from AO Foundation.

Whenever feasible, bone fragments should be preserved; when removed, they should be kept moist and organized on a simple sketch of the skull to maintain orientation for later reconstruction (Figure 6). After opening the sinus, the cavity is inspected for debris, foreign material, or mucosal damage before anterior table fragments are repositioned and fixed with microplates. Any defects larger than approximately 2–3 mm should be covered with mesh to minimize the risk of contour irregularity.

**Open Frontal Sinusotomy**

Open frontal sinusotomy is essentially the open analogue of an endoscopic Draf III procedure. This technique is employed when a large laceration or planned coronal incision provides adequate access, such as in cases with concomitant naso-orbito-ethmoid fractures. After exposing the frontal bone, all anterior table fragments are removed, kept moist, and laid out on a sketch of the defect for later replacement (Figure 6). A complete sinusotomy may be required to fully visualize the sinus cavity and outflow tract.

**Figure 7:**
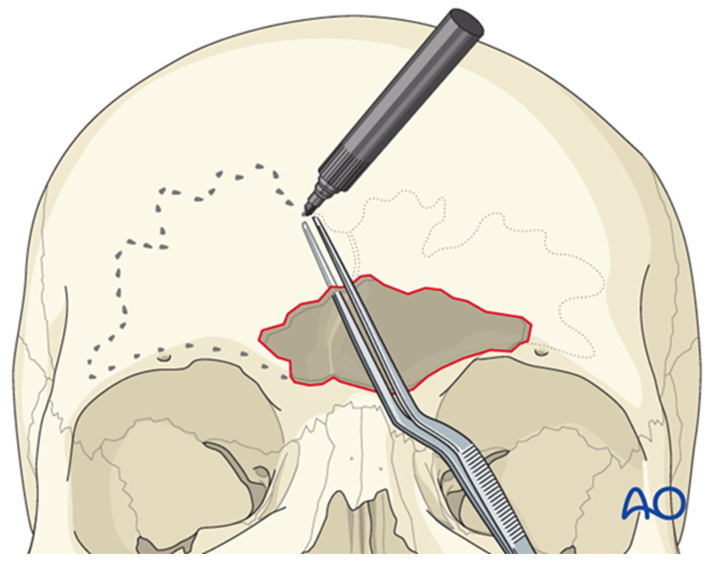
Bayonet forceps technique for delineating frontal sinus borders; the internal tine traces the sinus margin while the external tine guides external markings on the anterior table. Reproduced with permission from AO Foundation.

**Figure 8:**
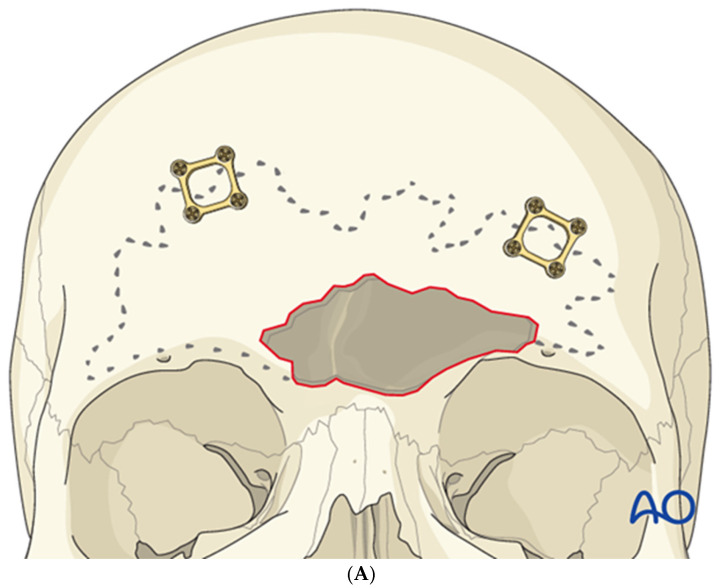

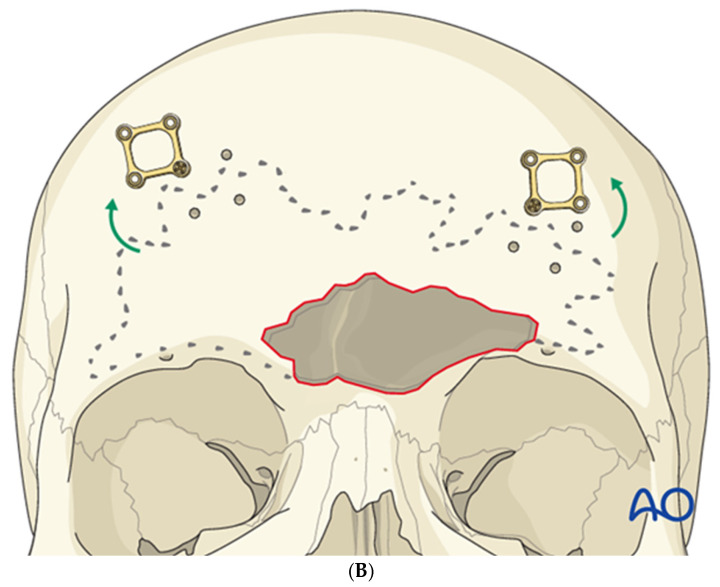
Thin plates spanning the sinus margins are pre-applied prior to osteotomy to aid accurate repositioning (**A**), then rotated away from the osteotomy line before cutting (**B**). Reproduced with permission from AO Foundation.

**Figure 9:**
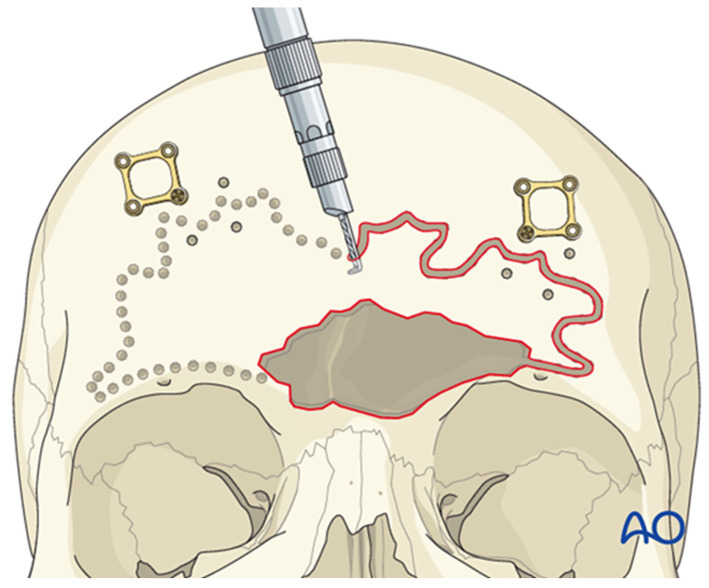
A side-cutting burr or saw is used to complete the osteotomy, allowing removal of the anterior table of the frontal sinus. Reproduced with permission from AO Foundation.

The sinus borders can be mapped using navigation or by employing bayonet forceps, with one tine inside the sinus and the other outside; as the internal tine is “walked” around the perimeter, markings on the outer table delineate the sinus margins (Figure 7). Thin plates are then applied across the planned osteotomy lines to facilitate accurate repositioning of the anterior table (Figure 8A). Each plate is rotated away from the osteotomy, leaving one screw in place (Figure 8B), and a side-cutting burr is used to complete the osteotomy (Figure 9). Care must be taken to preserve the supraorbital and supratrochlear neurovascular bundles, and a curved osteotome may be used to fracture the inter-sinus septum if necessary.

This approach allows direct access to the frontal outflow tract from above and is typically supplemented with endoscopic visualization from below. A rongeur is used to remove the inter-sinus septum, and a drill widens the outflow tract by removing the frontal beak and superior nasal septum, creating a single common outflow pathway. A silastic stent, packed with bioabsorbable material, is placed to maintain the sinusotomy, and the anterior table is reconstructed with microplates and micromesh for any residual gaps.

Postoperative care includes nasal saline mist and steroid irrigations, with stent removal at about three weeks and endoscopic surveillance to ensure long-term patency.

**Frontal Sinus Cranialization**

Cranialization is reserved for severe posterior table injuries or CSF leaks that cannot be managed endoscopically. The procedure is performed through a coronal incision, with removal of the anterior table as described for open frontal sinusotomy. All mucosal lining of the inner aspect of the anterior table must be drilled away, and in collaboration with neurosurgery, posterior table fragments are removed while remaining sinus edges are drilled down to the level of the anterior cranial fossa to minimize residual mucosa and reduce late mucocele risk.

**Figure 10:**
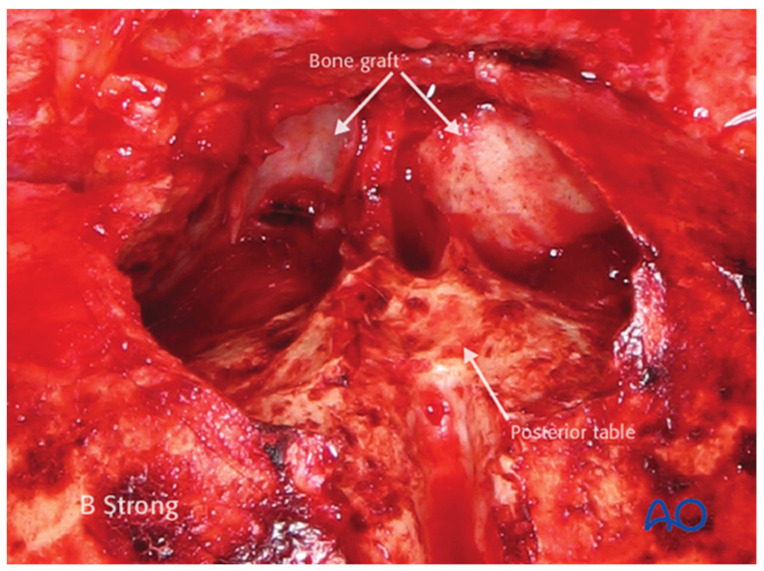
A temporalis muscle graft, followed by small bone grafts, is wedged into the outflow tract to achieve layered separation from the nasal cavity. Reproduced with permission from AO Foundation.

Any dural defects are repaired, and it is crucial to separate the nasal cavity from the intracranial space by obliterating the outflow tract. This is accomplished by inverting mucosa to block the tract, followed by placement of a small temporalis muscle graft and a wedge of bone (approximately 5 × 5 mm) to create a layered separation from the nasal cavity (Figure 10). When a pericranial flap is used intracranially, the bony kerf above the supraorbital rims must be widened to avoid compressing the flap during reconstruction. The anterior table is then reconstructed with microplates and mesh, and the incision is closed. The role of postoperative antibiotic prophylaxis remains controversial [11,21].

**Frontal Sinus Obliteration and Ablation**

The authors no longer routinely perform frontal sinus obliteration, preferring to maintain a functioning sinus through open or endoscopic frontal sinusotomy, which allows clinic-based endoscopic evaluation and lowers the risk of mucocele and other delayed complications.

Frontal sinus ablation (Riedel procedure) is reserved for extensive osteomyelitis or hardware infection [22]. This operation involves removal of the anterior table, portions of the superior orbital rim, and any involved bone or hardware, with the soft tissue envelope draped over the posterior table to eliminate the sinus space. Because of the extensive bony resection, significant cosmetic deformity can occur, sometimes necessitating secondary reconstruction with patient-specific implants or free tissue transfer.

**Endoscopic Frontal Sinusotomy (Draf III/Modified Lothrop)**

Transnasal endoscopic frontal sinusotomy (Draf III/Modified Lothrop) is widely used for inflammatory frontal sinus disease and has been adapted to manage traumatic injuries of the anterior and posterior tables, dura, and outflow tract. The procedure may be unilateral or bilateral depending on injury pattern and should be undertaken by surgeons with advanced endoscopic skills and experience in open frontal sinus trauma. In general, endoscopic approaches are preferred for selected fractures with accessible anterior table depression or isolated frontal sinus outflow tract obstruction, in the absence of severe comminution, posterior table displacement, or intracranial injury. In contrast, open procedures are reserved for more complex patterns that require direct reduction of comminuted segments, repair of posterior table and dural defects, or frontal sinus cranialization.

**Figure 11:**
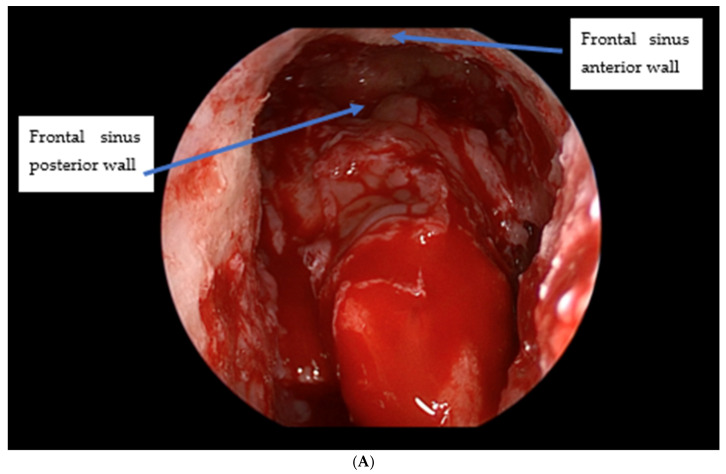

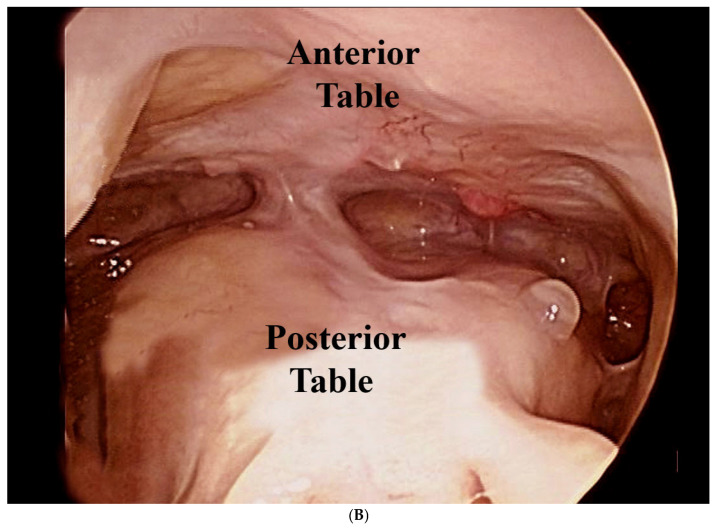
(**A**) Large horseshoe-shaped neo-ostium created after Draf III frontal sinusotomy, uniting both true ostia. (**B**) Well-healed frontal sinus outflow tract 10 months after repair.

The Draf III procedure involves removal of the agger nasi cell to expose the frontal outflow tract, followed by a 2 × 2 cm anterior septectomy to enable bilateral access. The mucosa anterior to the olfactory groove is elevated back to the first olfactory neuron, which defines the posterior limit of dissection. The frontal sinus is entered through the true ostium, and an endoscopic drill is used to remove the turbinate axilla and frontal process of the maxilla, exposing the periosteum of the nasal bones. The frontal sinus floor is progressively drilled away to create a large horseshoe-shaped neo-ostium uniting both natural ostia (Figure 11A).

Once the sinus cavity is fully visualized, traumatic defects of the anterior and posterior tables and skull base dura can be addressed. A silastic spacer filled with absorbable packing is positioned within the neo-ostium and left in place for about three weeks. Serial endoscopic examinations are necessary to detect outflow tract stenosis. Clinically significant stenosis of the frontal sinus outflow tract is less common after traumatic repair than in chronic inflammatory disease. Figure 11B illustrates a well-healed, patent outflow tract 10 months after injury as an example of this favorable long-term patency.

## 7. Complications

Complications may stem from the initial trauma or result from treatment. Early problems include wound infection, neurovascular injury (particularly to the facial nerve and supraorbital/supratrochlear nerves), and contour abnormalities. Infections are managed with antibiotics, facial nerve injuries are often observed or managed with chemodenervation with botulinum toxin of the contralateral side for improved symmetry, and most sensory deficits improve over time.

Late complications include persistent contour irregularities, chronic rhinosinusitis, mucoceles, and mucopyoceles, underscoring the need for long-term follow-up and surveillance imaging when indicated [23].

## 8. Summary

Frontal sinus fractures represent complex injuries that demand a systematic approach to evaluation and management. These high-energy injuries can cause sinus dysfunction, esthetic deformity, CSF leakage, mucoceles, and chronic sinusitis if not properly treated. Advances in imaging and operative techniques—especially endoscopic approaches—have resulted in a shift toward conservative, anatomically based strategies guided by the status of the anterior table, posterior table, and the nasofrontal outflow tract. Ongoing surveillance is essential to detect and treat delayed complications such as mucoceles, chronic infection, or evolving contour concerns [24,25]. We recognize that extended follow-up is difficult to achieve in craniomaxillofacial trauma populations, where nonattendance is common. In our service, we attempt to mitigate this by scheduling review appointments before discharge with clear written and verbal instructions, using reminder systems where available, and coordinating CMF follow-up with other trauma or rehabilitation visits to reduce the burden of additional hospital trips. For patients at highest risk of loss to follow-up, we also involve social work or case management to support attendance.

## Data Availability

The original contributions presented in this study are included in the article. Further inquiries can be directed to the corresponding author.
